# Inhibition of Atherosclerosis and Liver Steatosis by Agmatine in Western Diet-Fed apoE-Knockout Mice Is Associated with Decrease in Hepatic De Novo Lipogenesis and Reduction in Plasma Triglyceride/High-Density Lipoprotein Cholesterol Ratio

**DOI:** 10.3390/ijms221910688

**Published:** 2021-10-01

**Authors:** Anna Wiśniewska, Aneta Stachowicz, Katarzyna Kuś, Magdalena Ulatowska-Białas, Justyna Totoń-Żurańska, Anna Kiepura, Kamila Stachyra, Maciej Suski, Mariusz Gajda, Jacek Jawień, Rafał Olszanecki

**Affiliations:** 1 Chair of Pharmacology, Faculty of Medicine, Jagiellonian University Medical College, 31-531 Cracow, Poland; anna.niepsuj@uj.edu.pl (A.W.); aneta.stachowicz@uj.edu.pl (A.S.); katarzyna.1.kus@uj.edu.pl (K.K.); justyna.toton-zuranska@uj.edu.pl (J.T.-Ż.); a.kiepura@uj.edu.pl (A.K.); kamila.stachyra@uj.edu.pl (K.S.); maciej.suski@uj.edu.pl (M.S.); jacek.jawien@uj.edu.pl (J.J.); 2 Department of Pathomorphology, Jagiellonian University Medical College, 31-531 Cracow, Poland; magdalena.bialas@uj.edu.pl; 3 Department of Histology, Jagiellonian University Medical College, 31-034 Cracow, Poland; mariusz.gajda@uj.edu.pl

**Keywords:** atherosclerosis, fatty liver, agmatine, lipogenesis de novo, apoE-knockout mice

## Abstract

Atherosclerosis and NAFLD are the leading causes of death worldwide. The hallmark of NAFLD is triglyceride accumulation caused by an imbalance between lipogenesis de novo and fatty acid oxidation. Agmatine, an endogenous metabolite of arginine, exerts a protective effect on mitochondria and can modulate fatty acid metabolism. In the present study, we investigate the influence of agmatine on the progression of atherosclerotic lesions and the development of hepatic steatosis in apoE^−/−^ mice fed with a Western high-fat diet, with a particular focus on its effects on the DNL pathway in the liver. We have proved that treatment of agmatine inhibits the progression of atherosclerosis and attenuates hepatic steatosis in apoE^−/−^ mice on a Western diet. Such effects are associated with decreased total macrophage content in atherosclerotic plaque as well as a decrease in the TG levels and the TG/HDL ratio in plasma. Agmatine also reduced TG accumulation in the liver and decreased the expression of hepatic genes and proteins involved in lipogenesis de novo such as SREBP-1c, FASN and SCD1. In conclusion, agmatine may present therapeutic potential for the treatment of atherosclerosis and fatty liver disease. However, an exact understanding of the mechanisms of the advantageous actions of agmatine requires further study.

## 1. Introduction

Atherosclerosis is a chronic inflammatory disorder of the arterial vessels, often associated with dyslipidemia, characterized by endothelial dysfunction, infiltration of inflammatory cells into the vessel wall, an accumulation of lipids and fibrous elements, and the formation of atherosclerotic plaques [1]. The stability of the atherosclerotic plaque depends on the thickness of the fibrous cap and the degree of local cap inflammation [2]. The probability of plaque rupture, a catastrophic complication of coronary heart disease responsible for its clinical sequelae, acute coronary syndrome, is increased by the cap thinning, which is promoted by the enhanced activity of macrophages, death of vascular smooth muscle cells (VSMCs), and the breakdown of collagen and the extracellular matrix, which is marked by an increase in the volume of the necrotic core [3].

It is recognized that nonalcoholic fatty liver disease (NAFLD) is an early, independent risk factor for atherosclerosis development [4]. This complex liver disorder is prevalent worldwide, and covers a wide spectrum of pathological conditions ranging from simple hepatic steatosis to steatosis with inflammatory response—nonalcoholic steatohepatitis (NASH), which can develop into fibrosis, cirrhosis, and hepatocellular carcinoma [5]. It has been shown that increased caloric intake through the consumption of a high-fat, high-sugar diet, termed a Western diet, significantly contributes to obesity-related NAFLD [6]. From the point of view of metabolism, the excessive accumulation of triglyceride in the cytoplasm of hepatocytes, characteristic for NAFLD, depends on the imbalance between lipid uptake and de novo synthesis vs. fatty acid oxidation (FAO) and the export of lipids in the form of very low-density lipoprotein (VLDL) [7]. Mitochondrial dysfunction has also been shown to play a key role in the development of NAFLD and its progression to NASH [8]. In NAFLD, hepatic uptake and de novo lipogenesis (DNL) are increased, while a compensatory enhancement of mitochondrial fatty acid oxidation is insufficient [7]. Importantly, DNL seems to be the most important pathway that enables the accumulation of fatty acids in the liver under the condition of a Western diet [9,10]. The major controller of hepatic DNL is sterol regulatory element-binding protein 1c (SREBP-1c), which regulates the expression of all lipogenesis-associated enzymes [11]. Currently, there are no agents licensed to treat NAFLD, so dietary changes and increased physical activity to decrease body fatness remain the mainstream of recommended management strategies. However, such lifestyle changes are difficult to achieve for many patients [12,13].

Agmatine is an endogenous amine synthesized by decarboxylation of arginine in the presence of mitochondrial arginine decarboxylase (ADC). It can be formed in several mammalian tissues, especially in the brain, kidney or liver [14,15]. Agmatine is known to possess several interesting pharmacological properties: e.g., it is an agonist for α2-adrenergic and imidazoline receptors as well as an antagonist of NMDA receptors. It has been shown that agmatine has a protective effect on mitochondria along with anti-inflammatory and neuroprotective functions [16,17]. Such properties make agmatine a potential candidate for an agent to combat NAFLD. Agmatine affects hepatic metabolism including fatty acid oxidation, gluconeogenesis and glycolysis [18]. Our previous studies showed that exogenous agmatine inhibits atherosclerosis in apoE-knockout mice on a chow diet, which was associated with elevation of blood HDL-cholesterol and activation of β-oxidation of fatty acids in the liver [19]. While reports indicate that agmatine ameliorates atherogenesis in cholesterol-fed rabbits [20] and attenuates insulin resistance and in high-fructose-fed rats [21], its effect on liver steatosis has not been studied so far in animal NAFLD models.

Apolipoprotein E-knockout (apoE^−/−^) mice have been widely used in atherosclerosis research due to their propensity to spontaneously develop hypercholesterolemia and atherosclerotic plaques on a chow diet [22]. It has been shown that the apoE^−/−^ mice over 6 months of age on a chow diet develop mild hepatic steatosis, which is significantly exacerbated in mice on a high-fat diet (HFD) [23]. In this study we investigated the effects of agmatine on the progression of atherosclerotic lesions and the development of hepatic steatosis in apoE^−/−^ mice fed with a Western high-fat diet, with a particular focus on its effects on the DNL pathway in the liver.

## 2. Results

### 2.1. Body Weight

The mean body weight in the control (25.67 ± 1.78 g) and the agmatine-treated (26.22 ± 1.93 g) groups did not differ (*p* = 0.55). Additionally, food intake was similar in both groups.

### 2.2. Effects of Agmatine on Atherosclerotic Plaque

**Figure 1:**
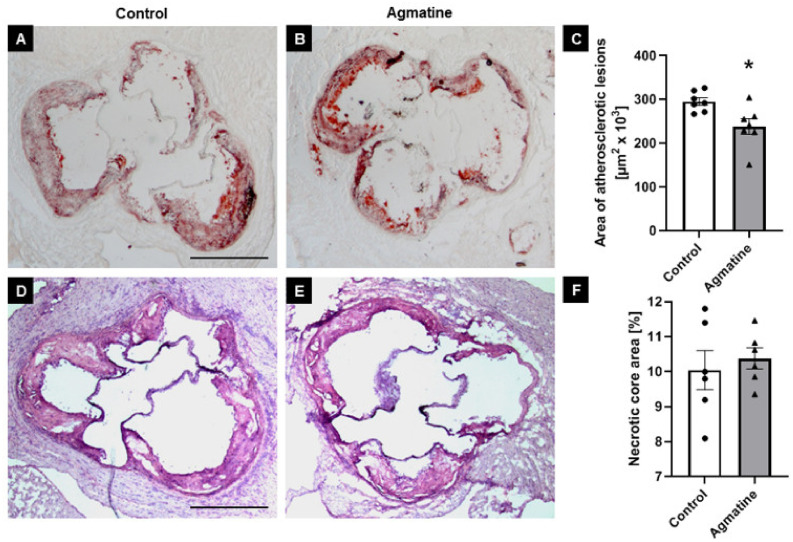
Influence of agmatine on atherosclerosis progression. Representative micrographs showing Oil Red O-stained atherosclerotic lesions (**A**,**B**) and HE-stained necrotic cores (**D**,**E**) in the aorta of control and agmatine-treated groups as well as their corresponding quantitative analyses (**C**,**F**). Magnification 40×. Scale bar = 500 μm. Data are mean ± SEM (* *p* < 0.05 as compared to control; *n* = 7 per group).

**Figure 2:**
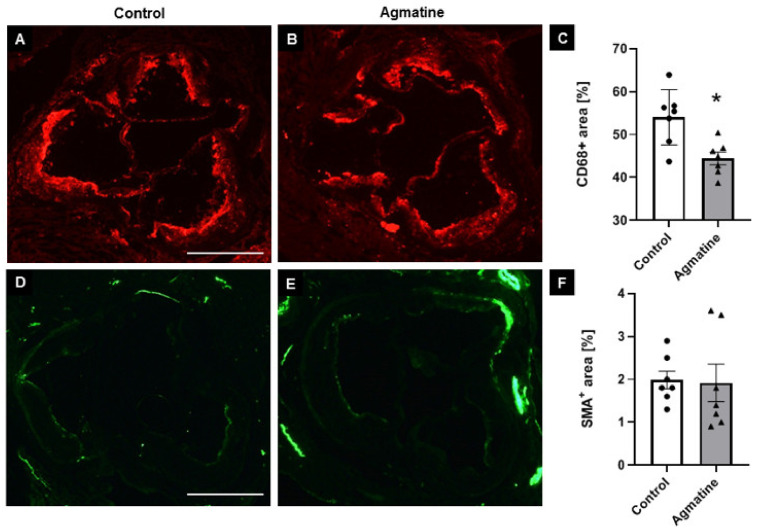
Impact of agmatine on the stability of atherosclerotic plaques. Immunohistochemical staining of aortic roots showing CD68-positive macrophages (**A**,**B**) and smooth muscle α-actin (SMA) (**D**,**E**) in control and agmatine-treated mice. Quantitative analysis of the atherosclerotic lesions area occupied by CD68-positive macrophages (**C**) and smooth muscle cells (**F**). Magnification 40×. Scale bar = 500 μm. Data are mean ± SEM (* *p* < 0.05 as compared to control mice; *n* = 7 per group).

To evaluate the impact of agmatine on the development of atherosclerosis, we treated apoE^−/−^ mice fed a Western high-fat diet with agmatine (20 mg per kg of body weight per day) for 16 weeks. The treatment with agmatine resulted in a significant decrease in atherosclerotic lesions in the aortas of apoE-knockout mice. Measured by the cross-section method, the area occupied by atherosclerosis lesions in the agmatine-treated group was 238,498 ± 6672 μm^2^, whereas in the control group it was 295,531 ± 3224 μm^2^ (Figure 1A–C). Agmatine did not have an influence on necrotic core formation in atherosclerotic plaque, as indicated by HE staining (control 10.1% ± 0.2% vs. agmatine 10.4% ± 0.1%) (Figure 1D–F). Agmatine administration significantly reduced the plaque area covered by CD68-immunopositive macrophages compared to the control group (44.5% ± 0.5% vs. 54.6% ± 0.8%) (Figure 2A–C), while the content of smooth muscle cells in the fibromuscular cap was similar in both groups (2.0% ± 0.3% vs. 1.9% ± 0.2%) (Figure 2D–F).

### 2.3. Effects of Agmatine on Lipid Profile in Serum

Agmatine treatment had an important influence on the lipid profile in apoE^−/−^ mice on Western HFD. The levels of serum TG were significantly lower by about 33% in the agmatine-treated group compared to the control animals. Agmatine showed only the tendency to decrease levels of TC and LDL and increase the levels of HDL (by about 30%). However, the ratio of TG/HDL showed a significant difference between groups (Table 1).

### 2.4. Effects of Agmatine on Liver Steatosis

**Figure 3:**
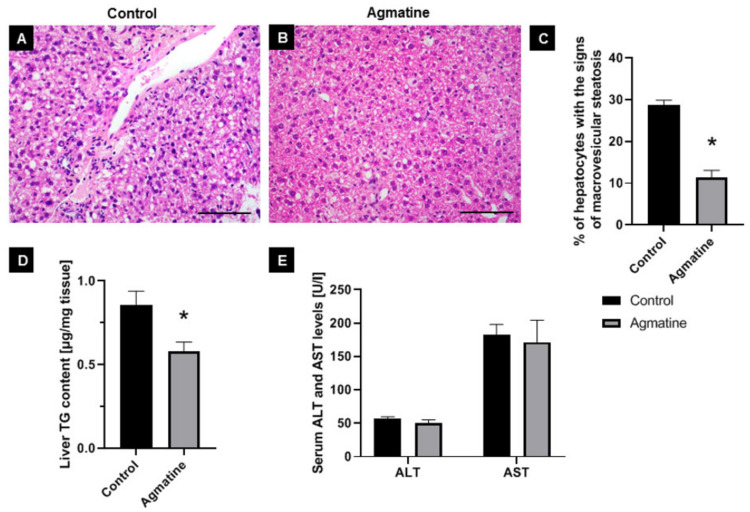
Influence of agmatine on the development of hepatic steatosis. Representative images of livers in control (**A**) and agmatine-treated mice (**B**). The figures show hematoxylin and eosin staining (**A**,**B**) and quantitative analysis of macrovesicular steatosis (**C**), triglycerides content in the liver (**D**) and the plasma ALT/AST levels (**E**) in control and agmatine-treated mice. Magnification 400× Scale bar = 50 μm. Data are mean ± SEM (* *p* < 0.05 as compared to control mice; *n* = 3–5 per group).

Hematoxylin-eosin staining showed changes in liver structure in both the control and agmatine-treated groups. In livers of control mice, the cytoplasm had a granular structure with signs of macrovesicular steatosis of about 30% of hepatocytes present in all three lobular zones. The lobular structure of the liver was still preserved and portal spaces were minimally enlarged, still with no inflammatory or necrotic changes. In livers of agmatine-treated mice, macrovesicular steatosis was found at about 10% of the hepatocytes, mostly in the first lobular area (around portal spaces). The lobular structure of the liver was still preserved and portal spaces were minimally enlarged, still with no inflammatory infiltrate. Small necrotic changes were noticed in the lobular areas (Figure 3A–C).

Moreover, treatment with agmatine resulted in a significant decrease in TG level of about 30% in the liver of apoE-knockout mice on Western HFD (Figure 3D). Plasma AST and ALT levels did not differ significantly between the control and agmatine-treated groups (Figure 3E).

### 2.5. Effects of Agmatine on Lipid Metabolism

**Figure 4:**
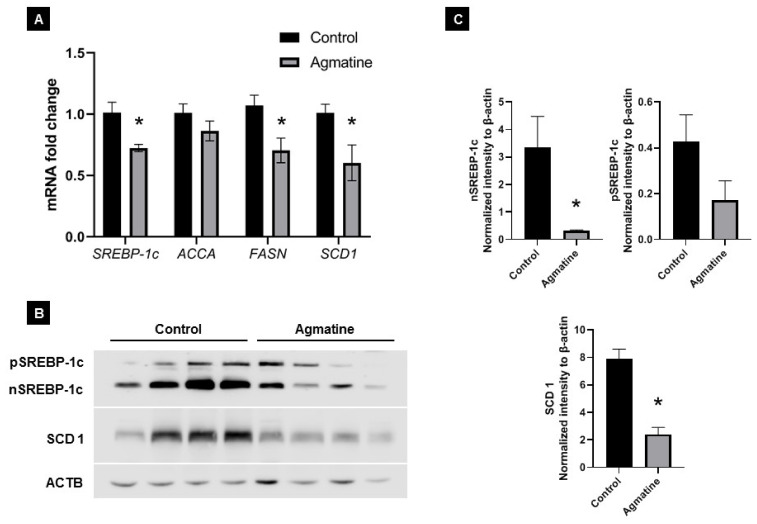
The mRNA expression of *SREBP-1c*, *ACCA*, *FASN* and *SCD1* in the liver of control and agmatine-treated mice (**A**). Validation of SREBP-1c, precursor and nuclear form, and SCD1 expression by Western blot (**B**,**C**). Data are mean ± SEM (* *p* < 0.05 as compared to control mice; *n* = 4–7 per group).

To investigate the molecular mechanisms responsible for the reduction in hepatic steatosis upon agmatine treatment of apoE^−/−^ mice on Western HFD, we evaluated the mRNA and protein expression of several molecules associated with lipogenesis in the liver. The *SREBP-1c*, *FASN* and *SCD1* mRNA levels were significantly decreased in the agmatine-treated group (0.72-, 0.70-, and 0.60-fold, respectively) compared with those in the control group (Figure 4A). The relative mRNA expression level of *ACCA* was also lower (0.86-fold) than those in the control group, but did not reach statistical significance (*p* = 0.22). Western blotting confirmed that the treatment with agmatine caused a significant decrease in nuclear, mature SREBP-1c and SCD1 protein levels (Figure 4B–D). The expression level of precursor SREBP-1c protein was also lower, but showing only a tendency towards significance (*p* = 0.15).

## 3. Discussion

Atherosclerosis remains the leading cause of death worldwide and NAFLD is associated not only with liver-related mortality per se, but it also poses a significant cardiovascular risk [24]. We have previously reported that prolonged administration of agmatine has antiatherosclerotic activity in apoE^−/−^ mice on a chow diet [19]. In this study, we demonstrated that agmatine significantly inhibited atherosclerosis and attenuated hepatic steatosis in more aggressive models of atherosclerosis and hepatic steatosis—in apoE^−/−^ mice fed a Western HFD. To our knowledge, this is the first report of agmatine inhibition of fatty liver in vivo.

NAFLD occurs with excess accumulation of triglycerides in the liver [25]. In this study, we showed that treatment with agmatine caused a 60% reduction in hepatocytes with signs of steatosis and a reduction in liver TG levels by 30%. Importantly, such effects were accompanied by significant decrease in the plasma TG levels. Such action by agmatine is particularly interesting in the context of the growing awareness of increased levels of plasma TG as a determinant of cardiovascular risk [26]. Interestingly, the use of agmatine in Western diet-fed apoE^−/−^ mice revealed differences in its effect on the plasma lipid profile. While the action of agmatine in mice on the chow diet mainly caused an insignificant increase in plasma HDL, under the conditions of a Western diet, its effect on the levels of TG appeared to be much stronger. Such a pattern of action by agmatine most likely depends on the fact that in the HFD models the dominant metabolic changes related to an increase in hepatic DNL and an increase in VLDL-TG secretion from hepatocytes [27]. Our results are also in line with other studies which demonstrate that agmatine treatment in rats fed a high-fructose diet inhibited hepatic steatosis, decreased liver glycogen content and reduced serum TGs, LDL and VLDL levels [21]. It has been proposed that the ratio of plasma TG to HDL may be a reliable indicator of metabolic and cardiovascular risk—its increase is associated with the exacerbation of insulin resistance, the occurrence of hypertension and an increase in residual cardiovascular risk [28,29,30]. It may well be that the beneficial influence of agmatine on lipid metabolism and plasma lipid profile represents an important mechanism of its anti-atherosclerotic action and a link between agmatine actions in the liver and vessel wall. In this context, a significant reduction in the TG/HDL ratio by agmatine in our model becomes a strong argument for further translational research.

Our results suggest that the effect of agmatine on the development of hepatic steatosis in Western HFD apoE^−/−^ mice is related to inhibition of DNL, as evidenced by the significant decrease expression of key factors and enzymes involved in this lipogenic pathway, such as: SREBP-1c, fatty acid synthase (FASN), and stearoyl-CoA desaturase 1 (SCD1). DNL begins from the conversion of citrate to acetyl-CoA by ATP-citrate lyase (ACLY), which is further turned into malonyl-CoA with the assistance of acetyl-CoA carboxylase (ACCA). Then, malonyl-CoA is condensed by fatty acid synthase (FASN) to produce palmitate, which finally turns to monounsaturated fatty acids, serving as the substrate of stearoyl-CoA desaturase 1 (SCD1) [10]. SREBP-1c plays a key role in lipid homeostasis by regulating the expression of genes involved in DNL and the synthesis of triglyceride [31]. SREBP-1c is a transcription factor which is synthetized in the endoplasmic reticulum as a precursor (125 kDa), which requires post-translational modification to yield its transcriptionally active nuclear form (65 kDa) [32]. In this study, agmatine caused the decrease in SREBP-1c in mRNA and protein expression, especially the active form of the latter. It was shown that activation of SREBP-1 is essential for development of hepatic steatosis [33]. Several studies have shown that SREBP-1 is upregulated in the livers of mice and patients with NAFLD [34]. The triglyceride levels are higher in transgenic mice with overexpression of SREBP-1c [35]. In addition, there is a correlation between SREBP-1c expression and the severity of insulin resistance. Interestingly, Sharawy et al. (2016) have shown that agmatine administration may attenuate insulin resistance through inhibiting SREBP-1c, mammalian target of rapamycin kinase (mTOR) and glucose transporter GLUT-2 in the liver of rats fed a high-fructose diet [21].

It is of note that in our study, agmatine also decreased the activity of the gene encoding the FASN, which was found to be overexpressed in NAFLD patients [36]. FASN catalyzes the last step in the synthesis of palmitate and is believed to be a major determinant of the maximal hepatic capacity to generate fatty acids by DNL [37]. Moreover, agmatine decreased the expression of SCD1 at both the mRNA and protein levels. SCD1 catalyzes the formation of monounsaturated fatty acids (MUFAs) from saturated fatty acids (SFAs). Intriguingly, SCD1 was shown to be involved in regulating diverse processes including inflammation, hormonal signaling, thermogenesis and both lipid synthesis and oxidation [38]. Although SCD1 is expressed ubiquitously, it is predominant in lipogenic tissues, especially hepatocytes and adipocytes [39]. It has been proposed that SCD1 may play an important role in TG accumulation in the hepatocytes; however, its precise role in NAFLD pathogenesis remains unclear [40]. Some studies show that the hepatic SCD1 expression may be increased by a high-fat diet [41], however in others its expression was decreased [42]. Interestingly, more studies confirm that SCD1 knockout mice are resistant to a high-fat- or high carbohydrate diet-induced steatosis [43,44]. The SCD1 knockout mice on HFD show decreased liver TG accumulation, reduced TG synthesis and increased fatty acid oxidation [45]. Moreover, MK-8245, an SCD1 inhibitor, has shown promising antidiabetic and antidyslipidemic efficacy in preclinical animal models [46]. Finally, Zhou et al. (2020) have recently suggested a novel function of SCD1 in hepatic lipogenesis, showing that the inhibition of SCD1 ameliorates hepatic steatosis by inducing AMPK-mediated lipophagy [47]. Altogether, our results indicate that agmatine in an animal model of NAFLD—apoE^−/−^ mice fed a Western diet—comprehensively inhibits the DNL pathway in the liver as well as beneficially alters the plasma lipid profile, and may be a promising drug candidate for prevention or treatment of fatty liver and atherosclerosis.

### Strengths and Limitations

Our research has several strengths: we conducted our research in a well-known model of atherosclerosis, of proven value in research on potential antiatherosclerotic drug candidates; we also used a model of fatty liver, based on the Western diet, which is highly caloric in nature, more relevant to the physiopathology of NAFLD than other models, e.g., based on lack of essential nutritional components; finally, we administered the tested compound by the noninvasive oral route at a dose that did not cause any side effects in mice. Nevertheless, our study also has limitations resulting from the need for extreme caution in transferring the results to the human situation, as well as the unquestionable need for further research into possible molecular mechanisms responsible for the action of agmatine, which is known to possess multiple pharmacological properties. It can stimulate α2-adrenergic and imidazoline receptors, block NMDA receptors and can accumulate in mitochondria and exert mitoprotective action. Thus, future studies should address which of the above mechanisms plays a role in the metabolic action of agmatine in the liver. In particular the role of agmatine in improving mitochondrial function needs to be addressed in the context of the pathophysiology of NAFLD.

## 4. Materials and Methods

### 4.1. Experimental Animals

Fourteen female apoE-knockout mice from the C57BL/6J background with average age of 6–8 weeks were purchased from Taconic (Ejby, Denmark). All animal procedures which have been performed conform to the guidelines from Directive 2010/63/EU of the European Parliament on the protection of animals used for scientific purposes and have been approved by the Jagiellonian University Ethical Committee on Animal Experiments (Krakow, Poland) (no. 67/2014).

### 4.2. Experimental Protocol

**Figure 5:**
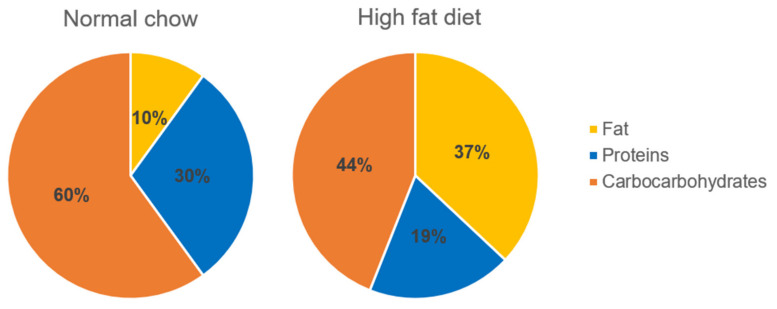
Percentage of energy sources obtained from diet; comparison of the normal diet with the Western high-fat diet used in this study.

The animals were housed in air-conditioned rooms (22.5 ± 0.5 °C, 50 ± 5% humidity) with 12 h light/12 h dark cycles, with free access to food and water, in the Animal House of Chair of Immunology of JUMC (Krakow, Poland). The mice were randomly divided into the control (*n* = 7) and the treatment (*n* = 7) groups. The mice in the control group were fed a Western high-fat diet (Western HFD diet, containing 15% fat + 0.25% cholesterol) (Labofeed B high-fat Diet, Wytwórnia Pasz Morawski, Kcynia, Poland). The percentage of energy obtained from this Western HFD compared to a normal chow is presented in Figure 5. In addition, the mice in the treatment group received a high-fat diet mixed with agmatine at a dose of 20 mg/kg of body weight per day. The dose of agmatine was chosen based on the previous results [19]. After 4 months of treatment, all mice were sacrificed using a carbon dioxide chamber in accordance with AVMA Panel 2007 recommendations and institutional IACUC guidelines. Blood samples were collected to prepare serum. The hearts were dissected, embedded in OCT compound (CellPath, Newtown, UK) and snap-frozen at −80 °C. Livers were removed and cut into 3 parts, including parts for TG determination, histological and real-time PCR analysis. Liver for histology was fixed in 4% formalin and for real-time analysis was stored in RNAlater (Ambion, Austin, TX, USA) at −80 °C.

### 4.3. Analysis of Atherosclerotic Plaque

The hearts with ascending aorta were sectioned (10 μm) for histological and immunohistochemical analysis, as described before [48]. To assess atherosclerotic lesions, sections were stained with Oil Red O (Sigma-Aldrich, St. Louis, MO, USA). The size of the necrotic core was measured on a hematoxylin-eosin (HE) staining according to a standard method. For immunohistochemistry, sections were processed using antibodies against CD68 (marker for macrophages, Serotec, Kidlington, UK) (dilution 1:800) and alpha smooth muscle actin (αSMA, Sigma-Aldrich, St. Louis, MO, USA) (dilution 1:800). Images were registered using an Olympus Camedia DP71 digital camera and analyzed using LSM Image Browser software (Zeiss, Jena, Germany).

### 4.4. Histology of the Liver

For hematoxylin-eosin (HE) staining, formalin fixed liver tissues were embedded into paraffin and cut into 2 μm sections. Samples were assessed microscopically for the presence of steatosis and the type of steatosis: microvesicular or macrovesicular. The mean percentage of steatotic hepatocytes has been specified in each case. Moreover, maintenance of lobular structure of the liver, the presence of inflammatory infiltrate both in lobular areas and in portal tracts and presence of necrotic changes of hepatocytes were described.

### 4.5. Biochemical Analysis

The blood was centrifuged for 10 min, 1000× *g* at 4 °C and the plasma was harvested and stored in -80 °C until use. The levels of total cholesterol (TC), triglycerides (TG), and low- and high-density lipoproteins (LDL and HDL) were measured using an enzymatic method on a Cobas 8000 analyzer (Roche Diagnostics, Indianapolis, IN, USA). In addition, plasmatic levels of aspartate aminotransferase (AST) and alanine aminotransferase (ALT) were determined by commercially available kits: Reflotron GPT and Reflotron GOT (Roche, Mannheim, Germany), and Reflovet Plus equipment (Roche, Mannheim, Germany).

TG levels in the liver were quantified using the Triglyceride Colorimetric Assay Kit (Cayman Chemical, Ann Arbor, MI, USA), according to the manufacturer’s protocol.

### 4.6. Real-Time PCR

Total RNA was isolated from the liver samples using ReliaPrep™ RNA Miniprep System (Promega, Madison, WI, USA), according to the manufacturer’s instructions. The concentration of RNA was determined by measuring the absorbance in an EPOCH Microplate Spectrophotometer (BioTek Instruments Inc., Winooski, VT, USA). The A260/A280 ratio was greater than 1.9 in all samples. The same amount of total RNA (1 μg) for each sample was reverse-transcribed using the High-Capacity cDNA Reverse Transcription Kit (Applied Biosystems, Foster City, CA, USA). Commercially available primers (*SCD1*, *SREBP1*, *FASN*, *ACCA* and *GAPDH*) from Bio-Rad (Hercules, CA, USA) and GoTaq^®^ qPCR Master Mix (Promega, Madison, WI, USA) were used to carry out the real-time PCR reaction. Analysis of relative gene expression with *GAPDH* as a reference gene was performed by the CFX96 Touch Real-Time PCR Detection System.

### 4.7. Western Blot

Samples containing equal amounts of total protein were mixed with gel loading buffer (50 mM Tris, 10% SDS, 10% glycerol, 10% 2-mercaptoethanol, 2 mg/mL bromophenol blue) in a ratio of 4:1 (*v/v*) and incubated at 95 °C for 5 min. Samples (30 μg of protein) were separated on SDS-polyacrylamide gels (12%) (Mini Protean II, Bio Rad, Hercules, CA, USA) using the Laemmli buffer system and proteins were semidry transferred to nitrocelulose membranes (GE Healthcare, Chicago, IL, USA). The membranes were blocked for 1 h with 5% (*w/v*) nonfat dried milk in TTBS and incubated overnight at 4 °C with specific primary antibodies: anti-SCD1 (1:500, R&D Systems, Minneapolis, MN, USA), anti-SREBP1 (1:1000, Novusbio, Littleton, CO, USA), anti-β-ACTIN (1:5000, Sigma, Saint-Louis, MO, USA) then for 1 h with HRP-conjugated secondary antibodies (GE Healthcare, Chicago, IL, USA). Bands were developed with the use of ECL-system reagents (GE Healthcare, Chicago, IL, USA). Rainbow markers (Amersham Biosciences, Amersham, UK) were used for molecular weight determinations. Protein pattern images were taken using an ImageQuant Las 500 (GE Healthcare, Chicago, IL, USA). Data analysis was performed using Image Lite Studio software (LI-COR, Lincoln, NE, USA).

### 4.8. Statistical Analysis

Data are expressed as mean ± standard error of measurement (SEM). Statistical analysis and graphing were carried out using GraphPad software Prism 9 (GraphPad Software Inc., San Diego, CA, USA). The equality of variance (F-test) and the normality of the data (Shapiro–Wilk test) were checked and then the nonparametric Mann–Whitney U test or *t*-test were used for statistical analysis of the data. Statistical significance was considered at *p* < 0.05.

## 5. Conclusions

**Figure 6:**
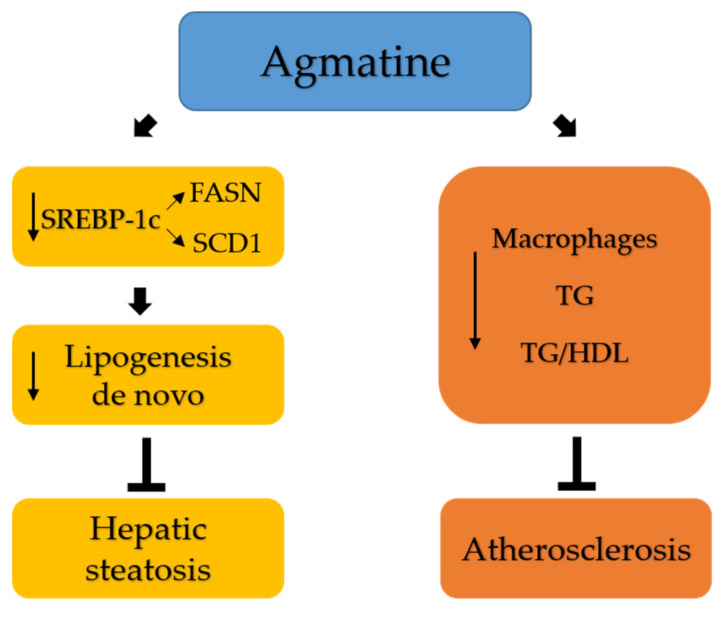
Graphical summary of the potential mechanisms of action of agmatine in atherosclerosis and hepatic steatosis. SREBP-1c, regulatory element-binding protein 1c; FASN, fatty acid synthase; SCD1, stearoyl-CoA desaturase 1; TG, triglycerides; TG/HDL, triglycerides/high-density lipoproteins. ↓ downregulation/decrease; ⏊ inhibition.

In summary, this study proves that agmatine inhibits the progression of atherosclerosis and attenuates hepatic steatosis by reducing the levels of triglyceride in the serum and in the liver of apoE^−/−^ mice on a Western high-fat diet. Such action is associated with the decrease in hepatic expression of genes involved in de novo lipogenesis. It is tempting to speculate that agmatine may provide a novel therapeutic approach to the treatment/prevention of atherosclerosis and fatty liver disease. However, the exact understanding of the mechanisms of the advantageous actions of agmatine require further study. The potential mechanisms of action of agmatine in atherosclerosis and hepatic steatosis are present in Figure 6.

## Figures and Tables

**Table 1:** Plasma lipid levels in control and agmatine-treated groups.

	TC (mmol/L)	LDL m(mol/L)	HDL (mmol/L)	TG (mol/L)	TG/HDL (mmol/L)
Control	28.01 ± 0.45	22.45 ± 0.45	3.61 ± 0.24	1.10 ± 0.09	0.32 ± 0.08
Agmatine	22.45 ± 0.53	19.71 ± 0.51	4.71 ± 0.21	0.74 ± 0.11 *	0.15 ± 0.04 *

Data presented as mean ± SEM; * *p* < 0.05 as compared to the control; *n* = 4 per group. TC—total cholesterol; LDL—low-density lipoproteins; HDL—high-density lipoproteins; TG—triglycerides; TG/HDL—triglycerides/high-density lipoproteins.

## Data Availability

All data are contained within this manuscript.

## Abbreviations

ACCA	Acetyl-CoA Carboxylase
ACLY	ATP-Citrate Lyase
ADC	Arginine Decarboxylase
ALT	Alanine Aminotransferase
AST	Aspartate Aminotransferase
DNL	De Novo Lipogenesis
FAO	Fatty Acid Oxidation
FASN	Fatty Acid Synthase
GADPH	Glyceraldehyde 3-Phosphate Dehydrogenase
HDL	High-Density Lipoproteins
HE	Hematoxylin/Eosin
HFD	High-Fat Diet
LDL	Low-Density Lipoprotein
NAFLD	Nonalcoholic Fatty Liver Disease
NASH	Nonalcoholic Steatohepatitis
NMDA	*N*-Methyl-D-Aspartate Receptor
RT-PCR	Reverse Transcription Polymerase Chain Reaction
SCD1	Stearoyl-Coa Desaturase 1
SMA	Smooth Muscle Actin
SREBP-1c	Sterol Regulatory Element-Binding Protein 1c
TC	Total Cholesterol
TG	Triglyceride
VLDL	Very Low-Density Lipoprotein
